# Reasons for discontinuing and restarting lithium multiple times: a case-register study based on the South London and Maudsley NHS Foundation Trust Clinical Record Interactive Search system

**DOI:** 10.1177/20451253251332275

**Published:** 2025-04-25

**Authors:** Petra Truedson, Kalliopi Vallianatou, Michael Ott, Martin Maripuu, Krister Lindmark, David M. Taylor, Ursula Werneke

**Affiliations:** Sunderby Research Unit, Department of Clinical Sciences/Psychiatry, Umeå University, Umeå, Sweden; Pharmacy Department, South London and Maudsley NHS Foundation Trust, London, UK; Institute of Pharmaceutical Sciences, King’s College London, London, UK; Department of Public Health and Clinical Medicine/Medicine, Umeå University, Umeå, Sweden; Department of Clinical Sciences/Psychiatry, Umeå University, Umeå, Sweden; Department of Public Health and Clinical Medicine/Medicine, Umeå University, Umeå, Sweden; Department of Clinical Sciences, Danderyd University Hospital, Karolinska Institutet, Stockholm, Sweden; Pharmacy Department, South London and Maudsley NHS Foundation Trust, London, UK; Institute of Pharmaceutical Sciences, King’s College London, London, UK; Sunderby Research Unit, Department of Clinical Sciences/Psychiatry, Umeå University, Umeå, Sweden

**Keywords:** bipolar disorder, drug-related side effects and adverse reactions, lithium, medication adherence, schizoaffective disorder

## Abstract

**Background:**

Despite the therapeutic benefits, non-adherence to lithium is common. One recent study showed that most patients discontinue lithium due to adverse effects. Little is known about individuals starting and discontinuing lithium repeatedly.

**Objectives:**

We aimed to determine reasons for discontinuing and restarting lithium multiple times in patients with bipolar or schizoaffective disorder.

**Design:**

Retrospective cohort study based on psychiatric case records of the SLaM Biomedical Research Centre Case Register (SLaM BRC case register).

**Method:**

Anonymised clinical data were extracted via the Clinical Record Interactive Search (CRIS) application. Patients with at least three events of lithium discontinuation between 2012 and 2022 were included.

**Results:**

Of 2888 eligible patients, 123 patients had discontinued lithium on at least three occasions. Psychiatric reasons, such as suspected lack of insight, feeling subjectively well or disagreeing with diagnosis, were the most common reasons for lithium discontinuations. They accounted for 77.2% of cases in the first event of discontinuation, 73.2% in the second and 72.3% in the third event. Adverse physical effects accounted for 19.5% of cases in the first event of discontinuation, 25.2% in the second and 26.0% in the third event. Relapse into the underlying affective disorder accounted for 83.7% each of reinstatements in the first and second events and 82.1% in the third event.

**Discussion:**

In our sample, lithium was discontinued due to adverse effects in only a minority of patients. In most cases, the reasons for lithium discontinuation were considered psychiatric. Lithium was mainly restarted due to relapse. This warrants a better understanding of the reasons for repeatedly discontinuing lithium and the best way to promote lithium adherence to prevent a perpetual cycle of remitting when on lithium and relapsing when off lithium.

## Introduction

### Background

Since the BALANCE trial published in 2010,^1^ lithium has experienced a revival as maintenance treatment for bipolar disorder (BD) in Europe. In 2014, the UK National Institute for Clinical Excellence (NICE) recommended lithium as a first-line, maintenance pharmacological treatment for BD.^2^ Yet, despite its therapeutic benefits, many individuals with BD may find lithium problematic to take long-term. Non-adherence to treatment is common in serious mental disorders, irrespective of the substance in question.^3^ For lithium, non-adherence rates vary from 14% to 68%.^4,5^ In a previous study conducted by our research group in the Northern Swedish region of Norrbotten, we found that of 873 patients treated with lithium, 54% had discontinued lithium on at least one occasion. Of all events of lithium discontinuation, 62% were due to adverse effects. However, this study mainly covered the first event of lithium discontinuation.^6^ The study did not explore in detail individuals who had discontinued and then restarted lithium repeatedly. Like any other drug, lithium may not work for everybody, and reports on response rates vary.^7–9^ Other individuals, as demonstrated in our previous work,^6^ may not be able to tolerate the adverse effects. Lack of insight and personal preference may also play a role in the decision to adopt or abandon lithium treatment. Additionally, a lifetime history of mixed episodes and alcohol use disorder may impair lithium effectiveness.^8^ There is a subgroup of individuals in whom the risk–benefit balance of lithium may be constantly changing, not being able to achieve an acceptable treatment outcome neither with nor without lithium. This group of individuals poses the greatest therapeutic challenge to clinicians; results from other studies cannot be extrapolated to this particular group. Yet, this group is rarely studied. To our knowledge, no study has been conducted into the reasons for discontinuing and restarting lithium multiple times.

### Aim/objectives

We set up a study to determine the reasons for discontinuing and restarting lithium multiple times. Specifically, we tested the following hypotheses for individuals having discontinued and restarted lithium multiple times:

1. Discontinuing lithium is mainly due to psychiatric reasons such as lack of perceived treatment effect and/or lack of insight into the underlying mental disorder rather than adverse physical effects.
2. Restarting lithium is mainly due to relapses into the underlying affective disorder requiring hospital admissions.
3. The time interval from restarting to discontinuing lithium becomes shorter with each reinstatement attempt.

## Methods

### Study design

We conducted a retrospective (historical) cohort study based on psychiatric case records to examine the reasons for discontinuing and restarting lithium multiple times. The study was conducted according to the STrengthening the Reporting of OBservational studies in Epidemiology (STROBE) checklist (Supplemental Appendix).^10^

### Setting

The study was conducted at the South London and The Maudsley NHS Foundation Trust Slam (SLaM). SLaM provides secondary mental health care to about 1.32 million people across the four London boroughs of Croydon, Lambeth, Lewisham and Southwark^11^ as well as tertiary and national specialist services to patients across the United Kingdom.^12,13^ Data for this study were obtained from SLaM’s Biomedical Research Centre Case Register (SLaM BRC case register), which extracts anonymised clinical data from SLaM’s electronic medical records via the Clinical Record Interactive Search (CRIS) application.^14^ SLaM BRC case register has been described in detail elsewhere.^14^ Since its development in 2008, CRIS has become a large-scale repository of anonymised clinical data. CRIS allows comprehensive access to de-identified records of patients cared by SLaM.^12^ CRIS has structured fields for demographic information and unstructured free text fields from case records and correspondence. The clinical information available in free text fields includes history, mental state examination, diagnostic formulation, medication and management plan.^15^

#### Data time frame

We included data from 1 January 2012, at which time the CRIS system was sufficiently developed to contain all relevant clinical information, to 31 December 2022.

### Participants

We included all individuals who (a) have had contact with SLaM, (b) were at least 18 years of age, (c) had received a primary diagnosis of either BD (F30 and F31) or schizoaffective disorder (SZD, F25) at any time according to the 10th revision of the International Statistical Classification of Diseases and Related Health Problems (ICD-10)^16^ and (d) had received lithium treatment but had discontinued on at least three occasions.

### Ethics and consent

The CRIS data resource has received ethical approval from The Oxford C Research Ethics Committee (Ref 08/H0606/71, 08/H0606/71+5, 18/SC/0372, 23/SC/0257). A patient-led oversight committee that provides governance for all projects conducted with CRIS approved the study. Additionally, ethical approval was obtained from the Swedish Ethical Review Authority (DNR 2022-05130-01).

### Outcomes

We examined the following outcome variables:

- Reasons for discontinuing lithium as recorded for the first and subsequent events.
- Reasons for restarting lithium as recorded for the first and subsequent events.
- Time interval from restarting to discontinuing lithium, with the first event of lithium reinstatement and the second event of lithium discontinuation as starting point.

### Exposures

We examined the following exposure variables: lithium treatment including times on and off lithium, number of discontinuations and reinstatements, diagnosis (BD or SZD) as established at the study endpoint, psychiatric comorbidities, sex, age when discontinuing lithium for the first time, ethnicity, person who took the initiative to discontinue and reinstate lithium (patient vs doctor), clinical setting at time of reinstatement (inpatient vs outpatient), other mood stabiliser(s) at time of reinstatement, last serum lithium (s-lithium) concentration within a year before discontinuation. The detailed definitions for these variables and the diagnostic procedures are given in Supplemental Appendix.

### Data extraction

The CRIS database was searched electronically with predefined algorithms to identify patients who had (a) an eligible diagnosis and (b) an indication of having received lithium treatment. All records were then hand-searched for evidence of lithium discontinuation and lithium reinstatement, using the keyword ‘lithium’ to identify relevant information. For each event, case records were then manually reviewed in detail to discover reasons for discontinuing and restarting lithium. When information was ambiguous or difficult to interpret, two members of the research team reviewed the free text together to achieve a consensus.

#### Reasons for lithium discontinuation

Reasons for lithium discontinuation were allocated to four categories, (a) psychiatric, (b) acute potentially life-threatening adverse effects, (c) non-acute physical adverse effects and (d) other reasons. Following the method of previous work by Öhlund et al.,^6^ non-adherence for psychiatric reasons included the following categories: refusing medication, feeling subjectively well, disagreeing with diagnosis, fear of developing adverse effects, not adhering to monitoring, uncertain effect and other psychiatric reasons. We also recorded as a psychiatric reason when there was no specific explanation given in the case records as to why lithium had been discontinued, but it was strongly suspected that this was due to the underlying psychiatric condition and/or lack of insight. Such suspicion for instance arose when patients had simply stopped taking lithium or had stopped having contact with SLaM without having been re-referred elsewhere.

#### Reasons for lithium reinstatement

Reasons for lithium reinstatement were defined in terms of relapse in the underlying affective disorder or fear of relapse, warranting lithium restart. We categorised relapses into manic, hypomanic, depressive, psychotic, mixed or catatonia.

### Control for bias and missing data

Automated extraction was used to ensure that all relevant patients were identified for potential inclusion. With the help of highlighted keywords, we then manually searched all free text fields derived from the anonymised case records to minimise the risk of missing events of discontinuing and restarting lithium. The study was limited to patients who were treated in secondary or tertiary care, that is, SLaM specialist psychiatric services. Some patients had parts of their treatment in primary care. For these, we could only rely on information mentioned explicitly in SLaM records. Hence, s-lithium concentrations taken in primary care were only accessible when explicitly mentioned in the SLaM records.

### Statistical analysis

We conducted a descriptive analysis, establishing the frequency of all outcome and exposure variables in our database. For each event of lithium discontinuation, that is, first, second, third or subsequent, we determined the mean s-lithium concentration within 1 year before lithium discontinuation. For each event, we also determined the time interval between restarting and discontinuing lithium.

## Results

### Baseline characteristics

**Figure 1.**
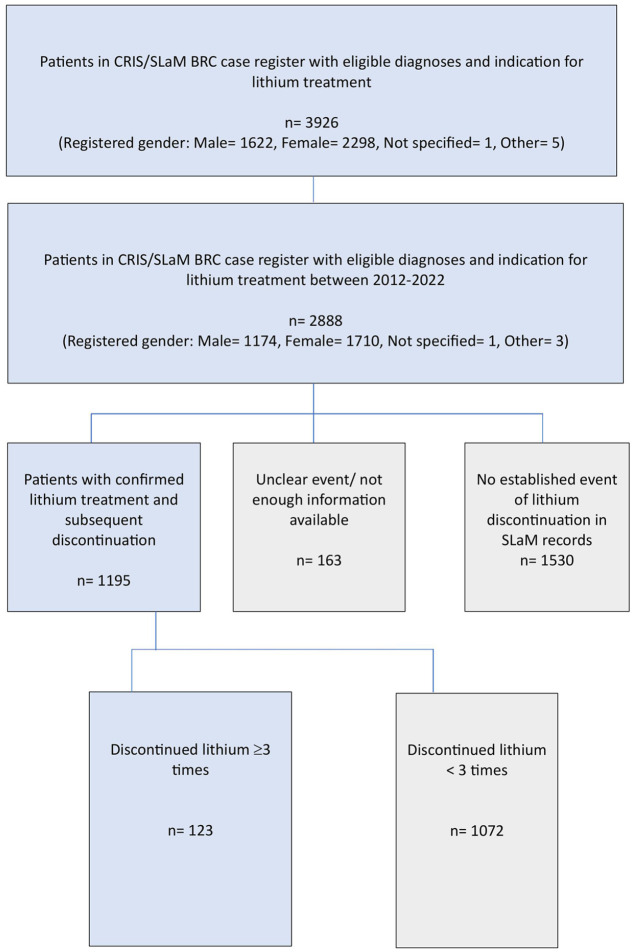
Selection of study sample.

There were 2888 patients eligible for inclusion with a diagnosis of BD or SZD and indication for lithium treatment between 2012 and 2022. Of these, 1174 (40.6%) were male, 1710 (59.2%) were female, 1 not specified and 3 other. We included 123 patients with at least three events of lithium discontinuation (Figure 1). For these 123 patients, 490 events of lithium discontinuations had been recorded between 2012 and 2022. Forty-seven (38.2%) were male and 76 (61.8%) were female. Fifty-six (45.5%) were White, 48 (39.0%) were Black, 7 (5.7%) Asian and the remaining 12 (9.8%) had mixed or other ethnicities. Fifty-nine (48.0%) patients had discontinued lithium three times and 64 (52.0%) patients had discontinued lithium at least four times. One patient had discontinued lithium 12 times. Ninety-eight (79.7%) patients had BD-1 or SZD; 25 (20.3%) patients had BD-2/other BD diagnosis. Twenty-nine (23.6%) patients had at least one psychiatric comorbidity, with personality disorder being most common in 18 (62.1%) patients. The median age for the first event of lithium discontinuation was 39.8 (min 18, max 78) years. The mean time until discontinuing lithium for the first time was 2.9 (SD 3.8) years. The median time until discontinuing lithium for the first time was 1.2 (min 0, max 17) years. At endpoint of the study, 47 (38.2%) patients had restarted lithium (Table 1).

**Table 1.** Baseline characteristics in individuals having discontinued lithium at least three times.

Baseline characteristic	⩾3 Events of discontinuation (*n* = 123)
Sex, *n* *(%)*	
Female	76 (61.8)
Male	47 (38.2)
Ethnicity, *n* *(%)*	
White	56 (45.5)
Black	48 (39.0)
Asian	7 (5.7)
Mixed	4 (3.3)
Other	8 (6.5)
Type of affective disorder, *n* *(%)*	
BD-1	49 (39.8)
SZD	49 (39.8)
BD-2/other BD	25 (20.3)
Age at first event of lithium discontinuation, years	
Median (min–max)	39.8 (18–78)
Psychiatric comorbidities, *n* *(%)*^a^	29 (23.6)
Personality disorder	18 (14.6)
Substance use/disorder	15 (12.2)
Other	5 (4.1)
Patients discontinuing lithium repeatedly, *n* *(%)*	
3 events	59 (48.0)
4 events	35 (28.4)
5 events	15 (12.2)
6 events	9 (7.3)
7 events or more	5 (4.1)
Time until first lithium discontinuation, years	
Mean (SD)	2.9 (3.8)
Median (min–max)	1.2 (0–17)
Lithium status at end of study (31 December 2022), *n* *(%)*	
On lithium	47 (38.2)
Off lithium	76 (61.8)

a Patients may have had more than one comorbidity at the same time. Cf. appendix for definition.

BD, bipolar disorder; max, maximum; min, minimum; *n*, number; SD, standard deviation; SZD, schizoaffective disorder.

### Characteristics of lithium discontinuations

For all events of lithium discontinuation, irrespective of whether first or any subsequent event, psychiatric causes were the most common reason for lithium discontinuation with more than 70% for each event (Table 2). Specifically, psychiatric reasons accounted for 95 (77.2%) cases in the first event of lithium discontinuation, 90 (73.2%) in second and 89 (72.3%) in third event. Mostly these psychiatric causes were strongly suspected, but not further specified. Adverse physical effects (acute and non-acute) accounted for 24 (19.5%) cases in the first event of lithium discontinuation, 31 (25.2%) cases in the second and 32 (26.0%) cases in the third event. On individual level, reasons recorded for the first five events of lithium discontinuation varied widely (Table 2). From the sixth event of lithium discontinuation, the reasons were mainly psychiatric (Table 2). Up to the fourth event, some patients had several main reasons for lithium discontinuation. Thereafter, patients had only one main reason for lithium discontinuation (Table 2). The 9th to the 12th event of lithium discontinuation only applied to two patients.

**Table 2.** Reasons for lithium discontinuation.

Event of lithium discontinuation	1st	2nd	3rd	4th	5th	6th	7th	8th	9th	10th	11th	12th
Patients with event, *n*	123	123	123	64	29	14	5	3	2	2	1	1
Reasons, *n*	128	128	127	69	29	14	5	3	2	2	1	1
Events with >1 reason, *n* *(%)*	5 (4.0)	3 (2.4)	4 (3.3)	5 (7.8)	—	—	—	—	—	—	—	—
Psychiatric reasons, *n* *(%)*^a^												
Strongly suspected but not further specified	63 (51.2)	61 (49.6)	60 (48.8)	33 (51.6)	16 (55.2)	9 (64.3)	2 (40.0)	2 (66.7)	2 (100.0)	2 (100.0)	—	—
Refusing medication	5 (4.1)	4 (3.3)	7 (5.7)	2 (3.1)	1 (3.4)	2 (14.3)	1 (20.0)	—	—	—	—	—
Feeling subjectively well	6 (4.8)	6 (4.8)	5 (4.1)	4 (6.3)	—	—	—	—	—	—	—	—
Not agreeing with diagnosis	5 (4.1)	1 (0.8)	1 (0.8)	1 (1.6)	1 (3.4)	—	1 (20.0)	—	—	—	—	—
Fear of developing adverse effects	10 (8.1)	10 (8.1)	8 (6.5)	4 (6.3)	3 (10.3)	—	—	1 (33.3)	—	—	1 (100.0)	1 (100.0)
Not adhering to monitoring	2 (1.6)	2 (1.6)	—	1 (1.6)	—	—	—	—	—	—	—	—
Uncertain effect	4 (3.3)	5 (4.1)	8 (6.5)	4 (6.3)	2 (6.9)	—	1 (20.0)	—	—	—	—	—
Other^b^	—	1 (0.8)	—	—	—	—	—	—	—	—	—	—
Psychiatric reasons, total	95 (77.2)	90 (73.2)	89 (72.3)	49 (76.6)	23 (79.3)	11 (78.6)	5 (100.0)	3 (100.0)	2 (100.0)	2 (100.0)	1 (100.0)	1 (100.0)
Acute potentially life-threatening adverse effects, *n* *(%)*^a^												
Voluntary or involuntary intoxications	—	3 (2.4)	4 (3.3)	1 (1.6)	—	—	—	—	—	—	—	—
Suspected NMS	1 (0.8)	—	—	1 (1.6)	1 (3.4)	1 (7.1)	—	—	—	—	—	—
Non-acute physical adverse effects, *n* *(%)*^a^												
Declined kidney function	6 (4.8)	1 (0.8)	10 (8.1)	2 (3.1)	1 (3.4)	—	—	—	—	—	—	—
Polyuria/polydipsia/diabetes insipidus	2 (1.6)	5 (4.1)	—	2 (3.1)	—	1 (7.1)	—	—	—	—	—	—
GI symptoms	—	4 (3.3)	4 (3.3)	2 (3.1)	—	—	—	—	—	—	—	—
Diarrhoea	1 (0.8)	—	—	—	—	—	—	—	—	—	—	—
Tremor	4 (3.3)	2 (1.6)	3 (2.4)	2 (3.1)	—	—	—	—	—	—	—	—
Stiffness	1 (0.8)	1 (0.8)	2 (1.6)	1 (1.6)	—	—	—	—	—	—	—	—
Sedation/numbness	5 (4.1)	2 (1.6)	1 (0.8)	2 (3.1)	1 (3.4)	—	—	—	—	—	—	—
Acne/skin condition	—	1 (0.8)	1 (0.8)	2 (3.1)	—	—	—	—	—	—	—	—
Weight gain	2 (1.6)	5 (4.1)	1 (0.8)	1 (1.6)	—	—	—	—	—	—	—	—
Other adverse effects^c^	2 (1.6)	7 (5.7)	6 (4.9)	1 (1.6)	1 (3.4)	—	—	—	—	—	—	—
Physical adverse effects (acute and non-acute), total	24 (19.5)	31 (25.2)	32 (26.0)	17 (26.6)	4 (13.8)	2 (14.3)	—	—	—	—	—	—
Other reasons, *n* *(%)*^a^												
Pregnancy or planned pregnancy	4 (3.3)	3 (2.4)	4 (3.3)	1 (1.6)	—	—	—	—	—	—	—	—
Other^d^	5 (4.1)	4 (3.3)	2 (1.6)	2 (3.1)	2 (6.9)	1 (7.1)	—	—	—	—	—	—
Other reasons, total	9 (7.3)	7 (5.7)	6 (4.9)	3 (4.7)	2 (6.9)	1 (7.1)	—	—	—	—	—	—

a Percentage calculated as reason(s)/patients with event.

b Change of mood stabiliser.

c Other adverse effects: incontinence; slurred speech; headache; ‘pain’; leg swelling; memory loss; ‘thyroid problems’; hair loss; hypernatraemia; anxiety; QT prolongation; request of naproxen which reduces lithium elimination; concerns about potentially reduced fertility.

d Medication error; stolen medication; can’t swallow pill; not specified.

GI, gastrointestinal; *n*, number; NMS, neuroleptic malignant syndrome.

**Figure 2.**
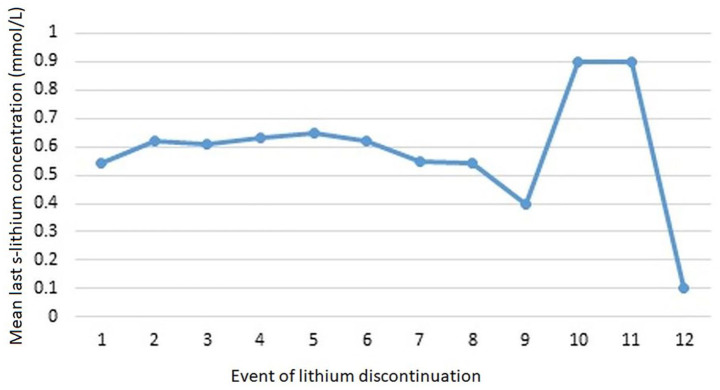
Mean last lithium serum concentration within 1 year before discontinuing lithium.

A s-lithium concentration taken within a year before discontinuation was available for most cases. This was irrespective of whether first or any subsequent event. The mean concentrations for the first, second and third events of lithium discontinuation were all in the lower therapeutic range, 0.5 (SD 0.2), 0.6 (SD 0.2) and 0.6 (SD 0.4) mmol/L, respectively (Figure 2). Details of s-lithium concentrations of all events and sample sizes are given in Supplemental Appendix.

### Characteristics of lithium reinstatements

Across all events, the most common reason for lithium reinstatement was relapse into the underlying affective disorder with 103 (83.7%) cases each in the first and second events and 69 (82.1%) in the third event. Manic relapses were most frequent with 65 (52.8%) cases in the first, 61 (49.6%) in the second and 37 (44.0%) in the third reinstatement. This was followed by depressive relapses with 15 (12.2%) cases in first, 18 (14.6%) in second and 14 (16.6%) in third event, and psychotic relapses with 15 (12.2%) cases in first, 16 (13.0%) in second and 9 (10.7%) in third event. Up to the fourth event of lithium reinstatement, some patients were restarted for fear of relapse warranting a mood stabiliser. From the fifth event of lithium reinstatement, this reason was not used (Table 3). Most patients were hospitalised at the time of reinstatement, 90 (73.2%) cases in the first, 82 (66.7%) in the second and 63 (75.0%) in the third event (Supplemental Appendix).

**Table 3.** Reasons for lithium reinstatement in individuals having discontinued lithium at least three times.

Reinstatement	1st	2nd	3rd	4th	5th	6th	7th	8th	9th	10th	11th
Patients with event, *n*	123	123	84	47	16	10	4	2	2	2	1
Manic episode, *n* *(%)*	65 (52.8)	61 (49.6)	37 (44.0)	16 (34.0)	9 (56.3)	8 (80.0)	2 (50.0)	—	—	1 (50.0)	—
Hypomanic episode, *n* *(%)*	8 (6.5)	7 (5.7)	6 (7.1)	2 (4.3)	—	—	1 (25.0)	—	—	—	—
Depressive episode, *n* *(%)*	15 (12.2)	18 (14.6)	14 (16.6)	9 (19.1)	3 (18.8)	1 (10.0)	—	1 (50.0)	1 (50.0)	1 (50.0)	1 (100.0)
Mixed episode, *n* *(%)*	—	—	1 (1.2)	1 (2.1)	—	—	—	—	—	—	—
Catatonic state, *n* *(%)*	—	1 (0.8)	2 (2.4)	2 (4.3)	—	—	—	—	—	—	—
Psychotic episode with no or minor affective symptoms, *n* *(%)*	15 (12.2)	16 (13.0)	9 (10.7)	8 (17.0)	3 (18.8)	1 (10.0)	1 (25.0)	1 (50.0)	1 (50.0)	—	—
No acute relapse but fear of relapse/need for mood stabiliser, *n* *(%)*	18 (14.6)	16 (13.0)	13 (15.5)	8 (17.0)	—	—	—	—	—	—	—
Other reasons^a^, *n* *(%)*	1 (0.8)	1 (0.8)	1 (1.2)	1 (2.1)	—	—	—	—	—	—	—
Unclear, *n* *(%)*	1 (0.8)	3 (2.4)	1 (1.2)	—	1 (6.3)	—	—	—	—	—	—

a Missed to renew prescription/dispense medication.

*n*, number.

#### Other mood stabiliser at time of reinstatement

At the time of lithium reinstatement, most patients were not treated with any other mood stabiliser. This applied to 71 (57.7%) cases in the first event, 60 (48.8%) in the second and 49 (58.3%) in the third event. If another mood stabiliser was prescribed at the time of reinstatement, this was predominantly a second-generation antipsychotic, either orally or as a long-acting injection (LAI). Combinations of at least two mood stabilisers were less common (Table 4).

**Table 4.** Other mood stabilisers used at the time of lithium reinstatement.

Reinstatement	1st	2nd	3rd	4th	5th	6th	7th	8th	9th	10th	11th
Patients with event, *n*	123	123	84	47	16	10	4	2	2	2	1
Mood stabiliser at time of lithium reinstatement, *n* *(%)*^a^											
None	71 (57.7)	60 (48.8)	49 (58.3)	24 (51.1)	11 (68.8)	5 (50.0)	2 (50.0)	1 (50.0)	1 (50.0)	1 (50.0)	1 (100.0)
Valproate	13 (10.6)	12 (9.8)	2 (2.4)	4 (8.5)	2 (12.5)	1 (10.0)	1 (25.0)	—	1 (50.0)	1 (50.0)	—
Lamotrigine	6 (4.8)	3 (2.4)	—	4 (8.5)	—	—	—	—	—	—	—
Other AE	3 (2.4)	3 (2.4)	1 (1.2)	—	—	—	—	—	—	—	—
SGA	40 (32.5)	41 (33.3)	11 (13.1)	12 (25.5)	3 (18.8)	4 (40.0)	1 (25.0)	1 (50.0)	1 (50.0)	1 (50.0)	—
SGA LAI	8 (6.5)	16 (13.0)	7 (8.3)	7 (14.9)	1 (6.3)	2 (20.0)	1 (25.0)	—	—	—	—
Unclear	2 (1.6)	4 (3.3)	4 (4.8)	2 (4.3)	—	—	—	—	—	—	—
Combinations of mood stabilisers used, *n* *(%)*											
No mood stabiliser	71 (57.7)	60 (48.8)	49 (58.3)	24 (51.1)	11 (68.8)	5 (50.0)	2 (50.0)	1 (50.0)	1 (50.0)	1 (50.0)	1 (100.0)
1 mood stabiliser only	33 (26.8)	44 (35.7)	21 (25.0)	17 (36.2)	4 (25.0)	3 (30.0)	1 (25.0)	1 (50.0)	—	—	—
⩾2 mood stabilisers, all oral	14 (11.4)	10 (8.1)	6 (7.1)	1 (2.1)	1 (6.3)	1 (10.0)	1 (25.0)	—	1 (50.0)	1 (50.0)	
⩾2 mood stabilisers including LAI^b^	3 (2.4)	5 (4.1)	4 (4.8)	3 (6.4)	—	1 (10.0)	—	—	—	—	—
Unclear	2 (1.6)	4 (3.3)	4 (4.8)	2 (4.3)	—	—	—	—	—	—	—

a Patients might have received more than one treatment at time.

b Including one event with a combination of olanzapine acute intramuscular injection and lamotrigine.

AE, antiepileptic drug; LAI, long-acting injection; *n*, number; SGA, second-generation antipsychotic, including clozapine.

**Figure 3.**
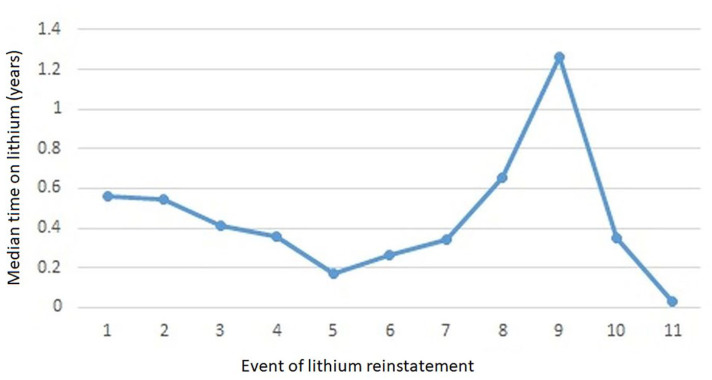
Median time on lithium treatment between lithium reinstatements and discontinuations.

The time interval between restarting and re-discontinuing lithium was a median of 0.56 (min 0, max 6) years between first lithium reinstatement and second lithium discontinuation, 0.55 (min 0, max 6) years between second lithium reinstatement and third lithium discontinuation, and 0.41 (min 0, max 6) years between third lithium reinstatement and fourth lithium discontinuation. Time on lithium treatment after fourth and fifth reinstatement was shorter. Thereafter, the treatment time between reinstatements and discontinuations fluctuated (Figure 3). The full details of the events and sample sizes are given in Supplemental Appendix.

### Circumstances around lithium discontinuation and reinstatement

**Figure 4.**
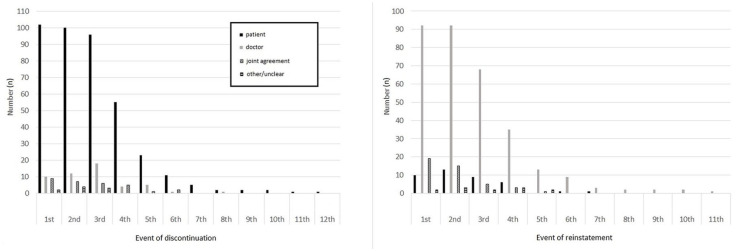
Agent taking initiative to discontinue and reinstate lithium.

There was a distinction as to who decided to discontinue and who decided to restart lithium. Across all events, it was mostly the patient who took the initiative to discontinue lithium, 102 (82.9%) cases in the first event of discontinuation, 100 (81.3%) in the second and 96 (78.0%) in the third event. However, it was mostly the doctor who decided to restart lithium, 92 (74.8%) each of cases in the first and second events and 68 (80.9%) in the third event of reinstatement (Figure 4). The full details of the events are listed in Supplemental Appendix.

## Discussion

### Summary of findings

To our knowledge, this is the first study investigating the reasons for repeated lithium discontinuations. Patients discontinuing and restarting lithium multiple times are particularly difficult-to-treat. This patient group is rarely explored. Although this group of patients is small in numbers, it has a great clinical need and can be expected to take up a disproportional amount of resources. In our study, 123 of 2888 (4%) patients had discontinued lithium at least three times during the 11-year observation time frame. Most of these patients (79.7%) had been diagnosed with BD-1 or SZD; in most of these, the reasons for lithium discontinuation were considered psychiatric. Only few patients discontinued lithium due to somatic adverse effects. Patients mostly took the decision to discontinue lithium and doctors mostly took the decision to restart lithium. Lithium reinstatement occurred mainly in the context of relapse into affective episodes requiring inpatient care. Black individuals were over-represented when compared with previous data regarding the SLaM cohort.^14^ Both sexes were equally represented when compared with the same cohort.

### Reasons for lithium discontinuations

The most common reasons for lithium discontinuations were considered psychiatric, irrespective of whether first or any subsequent event. We assumed that any lack of reason given pointed towards a psychiatric aetiology since in such events, somatic adverse effects were not flagged up explicitly. In the current study, we focused on a patient group that we considered particularly difficult-to-treat, having discontinued and restarted lithium repeatedly. Due to this selection, our study excluded a considerable group of patients having discontinued lithium for good after no more than two lithium treatment attempts. Therefore, our results cannot be extrapolated to the whole entity of patients who discontinue lithium. Hence, it is not surprising that the findings of our study contrast with older studies,^17–19^ in which adverse effects were reported as the most frequent reasons for lithium discontinuation. The results also contrast with a more recent study from Northern Sweden.^6^ In that study, adverse effects were also the most common reason for lithium discontinuation. As mentioned earlier, this study mainly concerned patients having discontinued lithium for the first time with limited information for patients having discontinued lithium repeatedly. A recent Canadian study examined lithium prescriptions of 14,008 patients between 2009 and 2018. Of these, 61.6% (CI 60.8–62.4) discontinued their lithium treatment at some point during the study. Users aged 18–24 were most likely to discontinue treatment.^20^ Lithium discontinuation occurred mostly in the first treatment year, irrespective of age or sex.^20^ Reasons for lithium discontinuation were not specified in this study. In a Dutch study of 135 geriatric patients, 49 patients (36.3%) had discontinued lithium. Of these, 25 patients had discontinued due to psychiatric reasons (including ‘non-compliance’) and 11 patients solely due to adverse effects. This study had a median follow-up of 18 months; most patients were treated for depression (57%).^21^ Regarding non-adherence to lithium, previous studies have reported different rates for non-adherence to lithium, ranging from 14% to 68%.^4,5^ Both doctors and patients may take the decision to discontinue lithium. According to a review by Gitlin, in terms of adverse effects, weight gain and cognitive dullness/impairment may particularly make patients discontinue lithium.^22^ Future work based on our current database, exploring patients having discontinued lithium for good after no more than two lithium treatment attempts could further expand the evidence base.

The reasons for discontinuing lithium multiple times warrant more investigation. In the current study, we were limited to information recorded in the psychiatric case records. But if patients had not communicated with psychiatric services before discontinuing lithium there would be no record. We suspect that lack of insight and severity of the affective disorder may have been most important in multiple discontinuers. Not achieving a sufficiently high s-lithium concentration may also have played a role; most patients in our study had s-lithium concentration at the lower end of the therapeutic range.

Establishing the reason for non-adherence with lithium can be difficult when a patient become acutely unwell and admitted to the hospital. However, at time of discharge, there is a risk that clinicians neither address nor document the reasons having led to admission. Then, an opportunity is missed to understand and formulate a patient’s specific needs regarding relapse prevention. Our findings highlight the importance of rethinking mood stabiliser maintenance treatment for patients who are not able or willing to adhere to lithium to avoid a perpetual cycle of relapses and remissions. Current guidelines, such as NICE and the Canadian Network for Mood and Anxiety Treatments (CANMAT), do not address this patient group as a separate entity.^2,23^

### Non-adherence

The World Health Organization has defined adherence ‘as the extent to which a person’s behaviour – taking medication, following a diet and/or executing lifestyle changes, corresponds with agreed recommendations from a health care provider’.^24^ Non-adherence may take different forms, not starting treatment, discontinuing treatment prematurely or not taking treatment according to the instructions.^25,26^ The underlying reason for non-adherence may be related to not understanding the treatment or not believing in the need of the treatment.^25^ Not understanding the treatment can arise from impaired cognition. Not believing in the need for the treatment can arise from lack of insight. Suboptimal or deficient communication and psychotic symptoms can affect both.^25–27^ Medication adherence and level of insight have indeed been shown to correlate.^28^ A recent study suggests that poorer insight seems to correlate with manic symptoms and decreased cognitive performance in patients with BD.^29^ As repeated relapses adversely affect prognosis, improving insight to extend symptom-free intervals is crucial.^30,31^ However, patients may consider some adverse effects such as diarrhoea and polyuria too embarrassing to report. Future studies could use in-depth interviews and cognitive testing to further explore the phenomenon of non-adherence and its contributing factors.

### Reasons for restarting lithium

We found that restarting lithium was mainly due to relapses into the underlying affective disorder, requiring hospital admissions in the majority of cases. Whereas patients mostly took the decision to discontinue lithium, doctors mostly took the decision to restart lithium. This finding is not surprising. Relapses into affective episodes particularly in the context of BD-1 or SZD often require inpatient treatment. Compulsory care is common when affective relapses occur in the form of manic episodes.^9^ Indeed, in our study, manic relapses were the most common presentation at the time of reinstatement. As mentioned previously, doctors may reinstate lithium because they wish to adhere to current guidelines such as NICE.^2^ However, guidelines often address ideal-world rather than real-world scenarios.^32^ If there is a history of multiple events of lithium discontinuation, the question arises of whether lithium is the best option after all. For instance, such patients might more likely benefit from LAI antipsychotics to achieve longer periods of affective stability. Once this has been achieved, patients may be in a better position to make informed decisions about psychopharmacological treatments. Lithium could then be reconsidered as a complement or possibly as an alternative.

### Lithium less effective when restarted?

Finally, the question arises of whether lithium loses effectiveness during subsequent treatment attempts.^33^ This could be an alternative explanation as to why patients repeatedly discontinue lithium. This could also account for ever-shorter time intervals between treatment attempts (examined in hypothesis three), with ever-shorter times on and off lithium reflecting an accelerated cycle of relapsing and remitting. We found one meta-analysis addressing this question in terms of number of relapses after restarting lithium. This meta-analysis did not show any increased risk for relapse after interruption of lithium treatment (OR 1.40, CI 0.85–2.31, *p* = 0.19). This finding should be interpreted with caution since this meta-analysis only included five relatively small studies with sample size between 28 and 130 patients.^34^ A recently published review by Kupka et al. investigating the original data of 403 patients suggests that there might be a subgroup in whom lithium discontinuation-induced treatment refractoriness exists.^35^ In this study, faster tapering of lithium, longer lithium-free interval after discontinuation, longer treatment duration before discontinuation and longer duration of BD before lithium start were associated with non-responsiveness.^35^ Our study was not designed to evaluate lithium effectiveness. We plan to explore the impact of having discontinued lithium on subsequent lithium treatment attempts in future work.

### Ethnicity

According to statistics from the UK National Health Service, no meaningful or reliable differences between sex and ethnic groups in terms of likelihood of screening positive for BD have been shown.^36^ However, Black individuals may experience a less favourable clinical course. This has been shown for psychotic disorders. In a previous SLaM study, among individuals with psychosis (including SZD), approximately 40% were of Black ethnicity.^37^ For common mental disorders, a survey from England has shown that individuals from any other ethnic group are less likely to receive effective treatment compared to White British people.^38^ Other studies have shown Black individuals to be more likely to receive LAI antipsychotics and less likely to receive psychotherapies.^39,40^ In our study, most patients were White. However, compared to a previously derived SLaM cohort profile,^14^ Black patients were over-represented with 39.0% against 24.7%.

### Strengths

Using SLaM BRC case register and CRIS provides access to large samples with detailed clinical information over long periods. In our study, we could assess 123 patients over 11 years with multiple events of lithium discontinuations. Using detailed information recorded in the anonymised case records allowed us to conduct an in-depth assessment of the actual circumstances regarding lithium discontinuation and reinstatement. Such detailed analysis would not have been possible with observational studies based on register data. Also, the information from the case records made it possible to establish the chronology of events, examine periods on and off lithium treatment and explore their relation to adverse effects.

### Limitations

The nature of our study was observational and retrospective. Relying on anonymised case records meant that the quality of our study depended on the quality of the information recorded. The quality of the case records was generally sufficient. However, in some cases, parts of the case records had been ‘blurred’ due to anonymisation, making it difficult to comprehensively follow the clinical course. Including only patients managed in secondary and tertiary care may have led to a bias towards more serious or more unstable clinical courses. It is also possible that we lost some patients due to lack of follow-up from SLaM, for instance when patients had been referred to primary care. These, however, would most likely re-enter secondary care, when becoming unstable. Some patients might have moved out of area and others might have deceased. As our CRIS search was guided by lithium exposure, we only had access to case records addressing lithium. In such cases, discontinuing lithium still would have been recorded. Finally, we did not distinguish between BD-1 and BD-2 disorders. As 80% of patients included had a diagnosis of BD-1 or SZD, this group seemed primarily affected. We judge our sample size to be too small to compare the different BD types meaningfully.

## Conclusion

In our sample, the reasons for patients who discontinued lithium multiple times were mostly considered psychiatric. Only a minority of patients discontinued due to adverse effects. Patients mainly restarted lithium due to relapse. In patients having discontinued lithium multiple times, the question arises of how best to promote adherence to treatment to prevent a perpetual cycle of remitting when on lithium and relapsing when off lithium. In such patients, switching to other mood stabilisers, including LAI antipsychotics, earlier rather than later may be indicated to prevent deterioration of prognosis due to multiple relapses. This scenario should be addressed in future research.

## Supplemental Material

sj-pdf-1-tpp-10.1177_20451253251332275 – Supplemental material for Reasons for discontinuing and restarting lithium multiple times: a case-register study based on the South London and Maudsley NHS Foundation Trust Clinical Record Interactive Search system Supplemental material, sj-pdf-1-tpp-10.1177_20451253251332275 for Reasons for discontinuing and restarting lithium multiple times: a case-register study based on the South London and Maudsley NHS Foundation Trust Clinical Record Interactive Search system by Petra Truedson, Kalliopi Vallianatou, Michael Ott, Martin Maripuu, Krister Lindmark, David M. Taylor and Ursula Werneke in Therapeutic Advances in Psychopharmacology

## Declarations

**Ethics approval and consent to participate:** The CRIS data resource has received ethical approval from The Oxford C Research Ethics Committee (Ref 08/H0606/71, 08/H0606/71+5, 18/SC/0372, 23/SC/0257). A patient-led oversight committee that provides governance for all projects conducted with CRIS approved the study. Additionally, ethical approval was obtained from the Swedish Ethical Review Authority (DNR 2022-05130-01). Since only already anonymised data were used according to the ethics approval granted, no consent to participate was obtained from the study participants.

**Consent for publication:** Not applicable since only already anonymised data were used according to the ethics approval granted.

**Competing interests:** P.T., K.V., M.M. and K.L.: None. U.W. has received lecture honoraria from Lundbeck and Janssen and has served/serves on scientific committees for Janssen and Teva, receiving honoraria for these activities. M.O. has been a scientific advisory board member for AstraZeneca AB, Sweden 2018–2020. D.T. has received research funding from Janssen, personal fees from Lundbeck, Otsuka, Viatris and Janssen and has received consultancy payments from Viatris, Merck and Teva. He holds shares in 428 Pharma, Myogenes and Saladax. David Taylor is Editor in Chief and Ursula Werneke an Associate Editor of Therapeutic Advances in Psychopharmacology and authors of this paper; therefore, the peer-review process was managed by alternative members of the board and the submitting editors have not been involved in the decision-making process.

**Availability of data and materials:** Data are owned by a third party, Maudsley Biomedical Research Centre (BRC) Clinical Records Interactive Search (CRIS) tool, which provides access to anonymised data derived from SLaM electronic medical records. These data can only be accessed by permitted individuals from within a secure firewall (i.e. the data cannot be sent elsewhere), in the same manner as the authors. For more information please contact: cris.administrator@slam.nhs.uk. The datasets generated and/or analysed during the current study are not publicly available due to lack of ethics committee permission. The coding specification and the structure of the dataset are available from the authors. Any other reasonable request will be raised with the healthcare provider.
