# Supplementary material for: Reasons for discontinuing and restarting lithium multiple times: a case-register study based on the South London and Maudsley NHS Foundation Trust Clinical Record Interactive Search system
Source: Ther Adv Psychopharmacol. 2025 Apr 25;15:20451253251332275. doi: 10.1177/20451253251332275 (PMC12035018; doi:10.1177/20451253251332275)
Supplement: sj-pdf-1-tpp-10.1177_20451253251332275 – Supplemental material for Reasons for discontinuing and restarting lithium multiple times: a case-register study based on the South London and Maudsley NHS Foundation Trust Clinical Record Interactive Search system [file sj-pdf-1-tpp-10.1177_20451253251332275.pdf]

Reasons for discontinuing and restarting lithium multiple times. A case-register study based on the South London and Maudsley NHS Foundation Trust Clinical Record Interactive Search (CRIS) System

Appendix

(Variable extractions and definitions, further detailed results, STROBE checklist)

Variable extractions and definitions

Automatic extraction: information extracted by the database administrators.
Manual extraction: information extracted from manual review of anonymised free text fields derived from the case records.

2.6 Exposures
Variable definitions:

Time on and off lithium: Manual extraction. If case records indicated that lithium was stopped or restarted a certain date, we selected that date as sufficiently accurate for lithium discontinuation or reinstatement. When the date was uncertain, we extrapolated the most likely date from the information in the case records. Estimation according to weeks, months, or years, depending on the accuracy of the recorded information re. lithium stop and re-start<br>• For years, we chose 1st July of any given year: 01-07-20XX<br>• For months, we chose 1st day of any given month: 01-XX-20XX<br>• For weeks within a month: 15th day within any given month: 15-XX-20XX
Diagnosis: Automatic extraction. BD-1, SZD, BD-2/other BD. All patients included in this study had received an ICD-10 diagnosis of either F30, F31 or F25 as a primary diagnosis on or after their 18th birthday. For diagnoses validation, we extracted all psychiatric ICD-10 F-diagnoses present during the observation time frame (2012-2022). The affective diagnoses were then manually validated. The main affective diagnosis was determined as either the most recorded affective diagnosis or, when diagnoses seemed to have changed during the observation time frame, the affective diagnosis that occurred most frequently at the end of observation time frame. For instance, an earlier BD-1 might have been reassessed as SZD. All patients who had at least one manic episode were considered to have BD-1 in line with DSM-5, unless a SZD diagnosis had been established.
Psychiatric comorbidities: Automatic extraction. Personality disorder, substance disorder/substance use, other. Manually validated according to ICD-10 F-diagnoses. When occurred on at least two occasions during the observation time frame (2012-2022). Occurred in the context of an underlying BD or SZD.
Sex: Automatic extraction. Male, female, not specified, other.
Age when stopping lithium for the first time: Automatic extraction and manual extraction. Years.
Ethnicity: Automatic extraction. White, Black, Asian, Mixed, Other.
Initiative to discontinue and reinstate: Manual extraction. We defined the person taking the initiative to stop and re-start lithium as either the patient, the doctor or as a joint agreement between them both. Any person other than patient or doctor was defines as "other".
Clinical setting at time of reinstatement: Manual extraction. Inpatient, outpatient, unclear.
Other mood stabiliser: Manual extraction. Use of other mood stabiliser(s) at time of reinstatement as described in the case records. We also recorded type of mood stabiliser and form of administration, oral or depot.
Last serum lithium concentration: Manual extraction. Lithium serum concentration within a year before lithium discontinuation.

BD: bipolar disorder; SZD: Schizoaffective disorder, ICD-10: International Classification of Diseases, 10th Revision; DSM-5: Diagnostic and Statistical Manual of Mental Disorders, Fifth Edition

Further detailed results

3.2 Characteristics of lithium discontinuations

Manual extraction. Full values for lithium concentrations.

Most recent lithium serum concentration in mmol/L taken within 1 year before lithium discontinuation

	Mean (SD)
1st discontinuation (n=123, 40 missing)	0.544 (0.244)
2nd discontinuation (n= 123, 32 missing)	0.617 (0.219)
3rd discontinuation (n= 123, 28 missing)	0.611 (0.352)
4th discontinuation (n= 64, 16 missing)	0.627 (0.273)
5th discontinuation (n=29, 3 missing)	0.649 (0.249)
6th discontinuation (n= 14, 6 missing)	0.624 (0.201)
7th discontinuation (n= 5, 1 missing)	0.547 (0.057)
8th discontinuation (n= 3, 0 missing)	0.543 (0.319)
9th discontinuation (n= 2, 1 missing)	0.4
10th discontinuation (n= 2, 1 missing)	0.9
11th discontinuation (n= 1)	0.9
12th discontinuation (n= 1)	0.1

n: number; SD: standard deviation

3.3 Characteristics of lithium reinstatements

Manual extraction. Full values for time between reinstatements and discontinuations.

Time in years between lithium reinstatements and lithium discontinuations

	Mean (SD)	Median (min-max)
1st reinstatement – 2nd discontinuation (n=123)	1.079 (1.277)	0.563 (0.005-6.436)
2nd reinstatement – 3rd discontinuation (n=123)	0.99 (1.256)	0.547 (0.002- 6.362)
3rd reinstatement – 4th discontinuation (n=64)	1.016 (1.324)	0.413 (0.019- 5.949)
4th reinstatement – 5th discontinuation (n=29)	0.505 (0.517)	0.358 (0.038- 2.614)
5th reinstatement - 6th discontinuation (n=14)	0.582 (0.836)	0.173 (0.002- 2.847)
6th reinstatement - 7th discontinuation (n=5)	0.458 (0.502)	0.265 (0.133- 1.352)
7th reinstatement – 8th discontinuation (n= 3)	1.507 (2.162)	0.342 (0.177- 4.002)
8th reinstatement - 9th discontinuation (n=2)	0.655 (0.489)	0.655 (0.309- 1.002)
9th reinstatement - 10th discontinuation (n=2)	1.266 (0.652)	1.266 (0.804- 1.727)
10th reinstatement - 11th discontinuation (n=1)	0.353	0.353
11th reinstatement- 12th discontinuation (n=1)	0.032	0.032

n: number; SD: standard deviation; min: minimum; max: maximum

Manual extraction. Clinical setting when lithium reinstatement.

Event of reinstatement	n	Inpatient setting	Outpatient setting	Unclear setting
1st	123	90	33	-
2nd	123	82	38	3
3rd	84	63	21	-
4th	47	32	14	1
5th	16	13	3	-
6th	10	9	1	-
7th	4	3	1	-
8th	2	1	1	-
9th	2	2	-	-
10th	2	1	1	-
11th	1	-	1	-

n: number

3.4 Circumstances around lithium discontinuation and reinstatement
Manual extraction. Initiative to discontinue lithium.

Event of lithium discontinuation	n	Patient	Doctor	Joint agreement between patient and doctor	Other/unclear
1st	123	102	10	9	2
2nd	123	100	12	7	4
3rd	123	96	18	6	3
4th	64	55	4	5	-
5th	29	23	5	1	-
6th	14	11	1	2	-
7th	5	5	-	-	-
8th	3	2	1	-	-
9th	2	2	-	-	-
10th	2	2	-	-	-
11th	1	1	-	-	-
12th	1	-	1	-	-

n: number

Manual extraction. Initiative to lithium reinstatement.

Event of reinstatement	n	Patient	Doctor	Joint agreement between doctor and patient	Other/unclear
1st	123	10	92	19	2
2nd	123	13	92	15	3
3rd	84	9	68	5	2
4th	47	6	35	3	3
5th	16	-	13	1	2
6th	10	1	9	-	-
7th	4	1	3	-	-
8th	2	-	2	-	-
9th	2	-	2	-	-
10th	2	-	2	-	-
11th	1	-	1	-	-

n: number

STROBE Statement—checklist of items that should be included in reports of observational studies

	Item No	Recommendation	Page No
**Title and abstract**	1	(*a*) Indicate the study's design with a commonly used term in the title or the abstract	1
		(*b*) Provide in the abstract an informative and balanced summary of what was done and what was found	2
**Introduction**			
Background/rationale	2	Explain the scientific background and rationale for the investigation being reported	3
Objectives	3	State specific objectives, including any prespecified hypotheses	3,4
**Methods**			
Study design	4	Present key elements of study design early in the paper	4
Setting	5	Describe the setting, locations, and relevant dates, including periods of recruitment, exposure, follow-up, and data collection	4
Participants	6	(*a*) *Cohort study*—Give the eligibility criteria, and the sources and methods of selection of participants. Describe methods of follow-up<br>*Case-control study*—Give the eligibility criteria, and the sources and methods of case ascertainment and control selection. Give the rationale for the choice of cases and controls<br>*Cross-sectional study*—Give the eligibility criteria, and the sources and methods of selection of participants	5
		(*b*) *Cohort study*—For matched studies, give matching criteria and number of exposed and unexposed<br>*Case-control study*—For matched studies, give matching criteria and the number of controls per case	N/A
Variables	7	Clearly define all outcomes, exposures, predictors, potential confounders, and effect modifiers. Give diagnostic criteria, if applicable	5,6
Data sources/ measurement	8*	For each variable of interest, give sources of data and details of methods of assessment (measurement). Describe comparability of assessment methods if there is more than one group	5,6
Bias	9	Describe any efforts to address potential sources of bias	6,7
Study size	10	Explain how the study size was arrived at	7,8
Quantitative variables	11	Explain how quantitative variables were handled in the analyses. If applicable, describe which groupings were chosen and why	7,19
Statistical methods	12	(*a*) Describe all statistical methods, including those used to control for confounding	7
		(*b*) Describe any methods used to examine subgroups and interactions	N/A
		(*c*) Explain how missing data were addressed	6
		(*d*) *Cohort study*—If applicable, explain how loss to follow-up was addressed<br>*Case-control study*—If applicable, explain how matching of cases and controls was addressed<br>*Cross-sectional study*—If applicable, describe analytical methods taking account of sampling strategy	N/A
		(*e*) Describe any sensitivity analyses	N/A

Continued on next page

**Results**			
Participants	13*	(a) Report numbers of individuals at each stage of study—eg numbers potentially eligible, examined for eligibility, confirmed eligible, included in the study, completing follow-up, and analysed	7
		(b) Give reasons for non-participation at each stage	8
		(c) Consider use of a flow diagram	8
Descriptive data	14*	(a) Give characteristics of study participants (eg demographic, clinical, social) and information on exposures and potential confounders	8
		(b) Indicate number of participants with missing data for each variable of interest	8
		(c) *Cohort study*—Summarise follow-up time (eg, average and total amount)	7,8
Outcome data	15*	*Cohort study*—Report numbers of outcome events or summary measures over time	8,9,10,11,12
		Case-control study—Report numbers in each exposure category, or summary measures of exposure	-
		Cross-sectional study—Report numbers of outcome events or summary measures	-
Main results	16	(*a*) Give unadjusted estimates and, if applicable, confounder-adjusted estimates and their precision (eg, 95% confidence interval). Make clear which confounders were adjusted for and why they were included	9,10,11,12
		(*b*) Report category boundaries when continuous variables were categorized	5,9,10,11,12
		(*c*) If relevant, consider translating estimates of relative risk into absolute risk for a meaningful time period	N/A
Other analyses	17	Report other analyses done—eg analyses of subgroups and interactions, and sensitivity analyses	N/A
**Discussion**			
Key results	18	Summarise key results with reference to study objectives	14
Limitations	19	Discuss limitations of the study, taking into account sources of potential bias or imprecision. Discuss both direction and magnitude of any potential bias	15,18
Interpretation	20	Give a cautious overall interpretation of results considering objectives, limitations, multiplicity of analyses, results from similar studies, and other relevant evidence	15,16,17
Generalisability	21	Discuss the generalisability (external validity) of the study results	15,18
**Other information**			
Funding	22	Give the source of funding and the role of the funders for the present study and, if applicable, for the original study on which the present article is based	20

**Give information separately for cases and controls in case-control studies and, if applicable, for exposed and unexposed groups in cohort and cross-sectional studies.*

Note: An Explanation and Elaboration article discusses each checklist item and gives methodological background and published examples of transparent reporting. The STROBE checklist is best used in conjunction with this article (freely available on the Web sites of PLoS Medicine at http://www.plosmedicine.org/, Annals of Internal Medicine at http://www.annals.org/, and Epidemiology at http://www.epidem.com/). Information on the STROBE Initiative is available at www.strobe-statement.org.

Source: https://www.equator-network.org/reporting-guidelines/strobe/. Accessed 28 January 2025.
